# High time resolution and high signal-to-noise monitoring of the bacterial growth kinetics in the presence of plasmonic nanoparticles

**DOI:** 10.1186/s12951-019-0459-1

**Published:** 2019-02-01

**Authors:** Marija Vukomanovic, Eduard Torrents

**Affiliations:** 1 Bacterial Infections: Antimicrobial Therapies, Institute for Bioengineering of Catalonia (IBEC), The Institute of Science and Technology, Baldiri Reixac 15-21, 08028 Barcelona, Spain; 2 Advanced Materials Department, Institute Jozef Stefan, Jamova 39, Ljubljana, Slovenia

**Keywords:** Presto Blue, Bacterial growth kinetics, Antimicrobial nanoparticles, Plasmonic nanoparticles, Arginine-functionalized gold

## Abstract

**Background:**

Emerging concepts for designing innovative drugs (i.e., novel generations of antimicrobials) frequently include nanostructures, new materials, and nanoparticles (NPs). Along with numerous advantages, NPs bring limitations, partly because they can limit the analytical techniques used for their biological and in vivo validation. From that standpoint, designing innovative drug delivery systems requires advancements in the methods used for their testing and investigations. Considering the well-known ability of resazurin-based methods for rapid detection of bacterial metabolisms with very high sensitivity, in this work we report a novel optimization for tracking bacterial growth kinetics in the presence of NPs with specific characteristics, such as specific optical properties.

**Results:**

Arginine-functionalized gold composite (HAp/Au/arginine) NPs, used as the NP model for validation of the method, possess plasmonic properties and are characterized by intensive absorption in the UV/vis region with a surface plasmon resonance maximum at 540 nm. Due to the specific optical properties, the NP absorption intensively interferes with the light absorption measured during the evaluation of bacterial growth (optical density; OD_600_). The results confirm substantial nonspecific interference by NPs in the signal detected during a regular turbidity study used for tracking bacterial growth. Instead, during application of a resazurin-based method (Presto Blue), when a combination of absorption and fluorescence detection is applied, a substantial increase in the signal-to-noise ratio is obtained that leads to the improvement of the accuracy of the measurements as verified in three bacterial strains tested with different growth rates (*E. coli*, *P. aeruginosa,* and *S. aureus*).

**Conclusions:**

Here, we described a novel procedure that enables the kinetics of bacterial growth in the presence of NPs to be followed with high time resolution, high sensitivity, and without sampling during the kinetic study. We showed the applicability of the Presto Blue method for the case of HAp/Au/arginine NPs, which can be extended to various types of metallic NPs with similar characteristics. The method is a very easy, economical, and reliable option for testing NPs designed as novel antimicrobials.
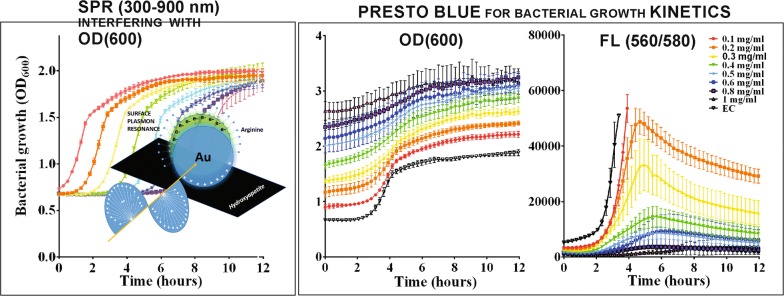

**Electronic supplementary material:**

The online version of this article (10.1186/s12951-019-0459-1) contains supplementary material, which is available to authorized users.

## Background

In light of the intensive, persistent, and progressive development of bacterial resistance to most of the available antimicrobial therapies including those in development or currently in use, the problem of antimicrobial multiresistance remains a hot topic and health concern worldwide [1–3]. Along with innovative strategies capable of improving antimicrobial treatment efficacy and reducing (eliminating) the possibility of bacteria developing antimicrobial resistance mechanisms, there is also a high demand for sophisticated methods capable of precise, reliable, and accurate antimicrobial activity determination and quantification [4]. Both directions, designing innovative antimicrobial strategies and designing reliable methods for monitoring their antimicrobial activity, are absolutely essential for making a sustainable plan for using antibiotics or new antimicrobials in the near future.

The Clinical and Laboratory Standards Institute (CLSI) [5] and the European Committee on Antimicrobial Susceptibility Testing (EUCAST) [6] recommend standardized and conventional susceptibility tests that include diffusion (i.e., Kirby–Bauer), dilution [i.e., determination of minimal inhibitory concentration (MIC), and minimal biocidal concentration (MBC) and a combination of these tests]. Presently, there are advanced methods designed for very precise monitoring of the antimicrobial activity [7–10] based on different modes of detection including optical (i.e., turbidity at 600 nm during spectrophotometric microplate readings) [11], fluorescence (i.e., following fluorescence markers in flow cytometry when Syto 9/propidium iodide is commonly used) [12] and molecular methods [i.e., quantitative polymerase chain reaction (qPCR) to detect and quantify the amount of target genomic material] [7]. These methods generally qualify as fast, comprehensive, and quantifiable ways to monitor bacterial activity and can follow the growth kinetics and differentiate between viable and nonviable cells [7]. Although their cost depends on the measuring device or equipment and the need for a labeling marker, they are highly suitable for large-scale and high-throughput screenings in research and industry.

However, despite the numerous benefits of advanced antimicrobial methods, some of these techniques are facing their limits regarding the testing of novel antimicrobials based on nanotechnology and new materials as clearly observed in previous studies [13, 14]. Presently, there is a very high diversity of NPs and nanostructures engineered for biomedical applications that are characterized by the alteration of various physicochemical properties induced by nanosizing [15, 16]. Switching to the use of NPs may contribute to antimicrobial activity of a material through the modifiable chemistry of their surface [17], changes in the surface-to-volume ratio [18], their variable shape and structural properties [19], their enhanced solubility and capacity for ion release [20, 21], etc. Because of their large surface area as well as high absorbability, reactivity, and catalytic activity, NPs can influence the signal measured during antimicrobial testing and provide an under- or over-estimation of their real antibacterial efficacy [22].

Due to their characteristic optical properties, some NPs (i.e., silver (Ag), gold (Au), copper and its oxide (Cu/CuO), zinc oxide (ZnO), gallium and its oxide (Ga/Ga_2_O_3_), magnesium oxide (MgO), carbon nanotubes (CNT), antibiotic-loaded degradable PLGA NPs, among others) [23, 24] are capable of nonspecific interactions (scattering, absorption, reflection, etc.) with light which influences/interferes with the signal detected during biological studies (like cytotoxicity or antimicrobial measurements) [13, 14]. These nonspecific interactions usually increase the background, decrease the signal-to-noise ratio, and affect the accuracy of the measurements. An excellent example is a study of Chen et al. [14] which observed a clear interference and formation of an intensive background while testing oxide NPs using the spectrophotometric approach by following the change in optical density (OD). As the authors report, the issue was resolved by counting colony forming units (CFU) or a flow cytometry (FCM) approach as alternative methods for assessing antimicrobial properties of the NPs tested. They found that FCM provided highly accurate results with high reproducibility despite increasing concentrations of NPs [14]. However, in contrast to OD (which was found to be less accurate), both of the other two methods, CFU and FCM, are limited to endpoint measurements and exclude the possibility of investigating the kinetics of bacterial growth in the presence of different NPs.

Endpoint measurements (i.e., bacterial viability or determination of the MIC and MBC) [25–28] obtained after exposure of bacteria to NPs during selected periods of time are usually more manageable. For endpoint measurements, there is usually a correction factor that is introduced to exclude the contribution of NPs to the measured signal. There is also a possibility of combining various detection methods so that optical detection is tested along an alternative testing approach (i.e., plating) to correlate and validate the obtained results.

In contrast to endpoint measurements, following the kinetics of bacterial growth in the presence of NPs is a much more difficult task and sometimes impossible. In some cases, the kinetics is investigated as a series of several endpoint measurements and usually includes sampling/staining in the middle of the kinetic study followed by long time intervals between two measurements. Examples are the 2-h interval applied for growth kinetics detection in chitosan-Ag-fluoride NPs using the Live/Dead BacLight Viability Kit [26] or the 30-min to 2-h intervals used for growth kinetics detection of MgO NPs using the 6 × 6 drop plate method [27]. Due to their low speed or low sensitivity, these methods do not allow for the required time resolution. More frequently, to obtain more accurate measurements (without any sampling steps during the kinetics study), the influence of NPs on the growth kinetics of bacteria is investigated by following the gradual change in the turbidity at 600 nm (OD_600_). When NPs are present, a change in the light absorption at 600 nm due to the bacterial growth is superimposed on nonspecific absorption by NPs (as observed for Ag NPs or other polymeric NPs) [28]. The problem is potentially resolved by introducing the correction factor (initial absorption of the medium with NPs and without bacteria). This can be efficient for testing highly stable NPs that are homogeneously distributed within the tested suspension, but that is usually not the case. More frequently, NPs aggregate locally and also precipitate and redisperse during testing which provides a nonhomogeneous change in the resulting turbidity in the tested suspension. Consequently, NPs may contribute substantially to the variation of the measured values thus increasing measurement errors and affecting the gathering of reliable and reproducible results.

Due to the need for more accurate bacterial growth studies and antimicrobial screenings in the presence of specific NPs, we conceptualized an innovative upgrade of a well-known resazurin-based method (Presto Blue) which can track the kinetics of bacterial growth at a high rate, with high time-resolution and without any additional steps (i.e., sampling) applied during the investigated process. For validation of the method we used a well- characterized, functionalized gold NPs (HAp/Au/arginine) [29], with specific optical properties, with demonstrated susceptibility in *Escherichia coli*, *Staphylococcus aureus*, and *Pseudomonas aeruginosa* PAO1. As we confirmed while testing arginine-functionalized gold in three different strains, the method provides accurate tracking of the bacterial growth in the presence of NPs and resolves the problem of intensive superposing of NP light absorption with OD_600_ measurements during regular turbidity-based investigations.

## Results and discussion

Resazurin-based dyes are routinely used to assess the viability of a range of biological systems including bacteria, yeast, fungi, protozoa, and eukaryotic cells [30–32]. Considering their capacity for very fast detection (10 min) with high sensitivity (12 cells per well for the commercially available dye Presto Blue [33]), we started our research with the aim of investigating the use of this dye for more accurate tracking of bacterial growth kinetics in the presence of antimicrobial NPs characterized by the ability to interfere with analytical techniques.

**Fig. 1:**
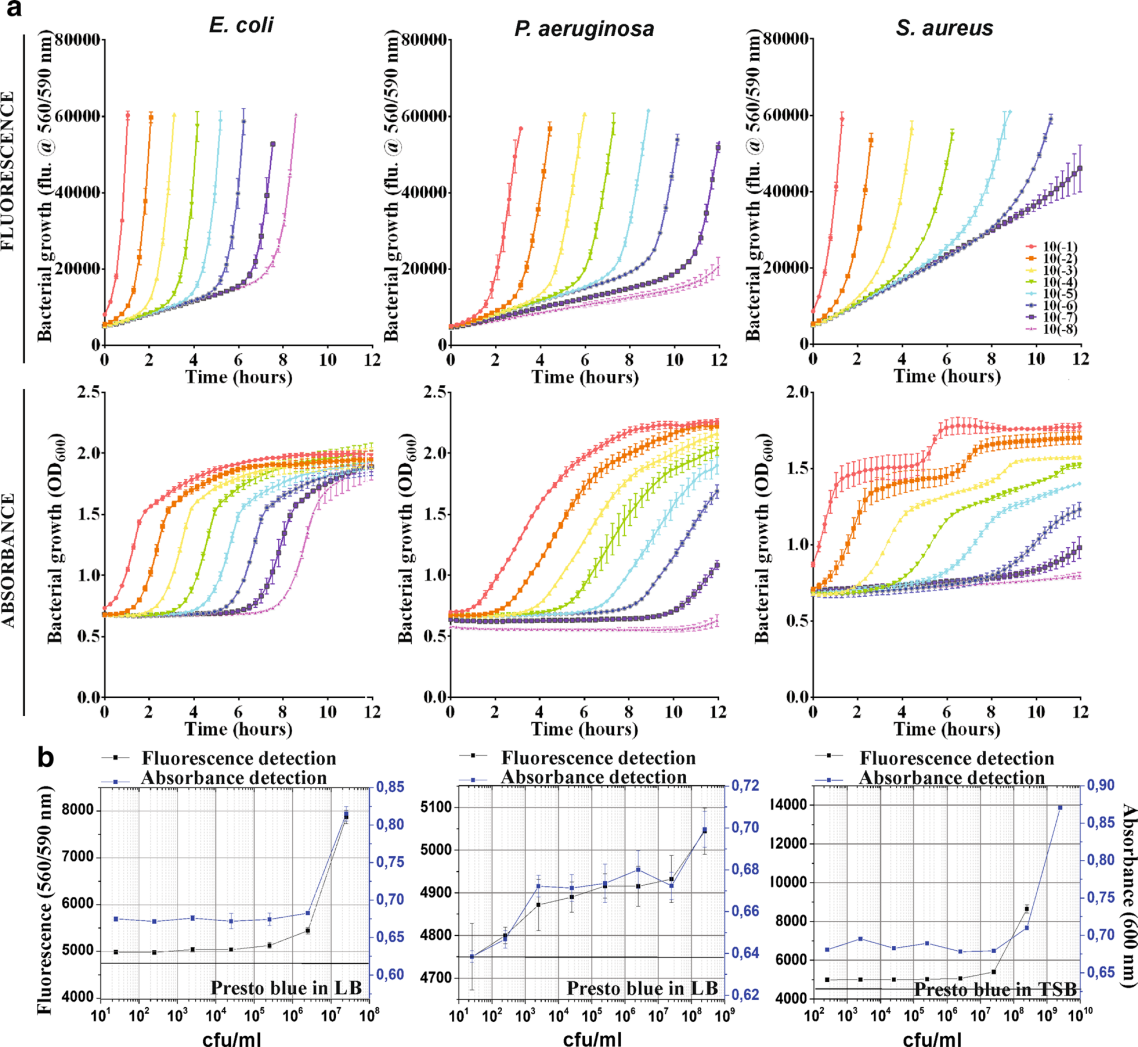
Growth curves kinetics (fluorescence and absorbance) (**a**) and correlation (**b**) of the absorbance (at 600 nm) and fluorescence (at 590 nm) detected after staining with Presto Blue for serial dilution of bacterial culture (cfu/ml) in the case of *E. coli*, *P. aeruginosa* and *S. aureus*

Initially, we performed a set of growth-kinetic studies for eight different bacterial concentrations using an overnight culture serially diluted from 10^−8^ to 10^−1^ (as indicated in the legend of Fig. 1). For the most optimal tracking of the bacterial growth, all samples contained a high content of dye added at the beginning of the monitoring process. Measurements were automatically performed every 15 min and included detecting an absorption signal at 600 nm followed by detecting the fluorescence signal with emission/excitation at 560/590 nm (Fig. 1a). The same type of experiment was repeated with three bacterial strains characterized by different bacterial growth kinetics, two Gram-negative (*E. coli* and *P. aeruginosa* PAO1) and one gram-positive (*S. aureus*). As shown in Fig. 1a, after both absorbance and fluorescence detection, the dye could track the bacterial growth kinetics at different bacterial concentrations for all three bacterial strains. A timeframe of 15 min set between two measurements was long enough to enable the detection of a change in bacterial growth and it provided a high time-resolution of the studied kinetic process. Although there was a frequent exposure to light during the measurement, the dye remained stable throughout the study and we did not observe any need for additional labeling. This can be attributed to the high concentration of the dye that was initially applied to the labeling procedure which provided a stable staining process throughout the 12-h period.

The critical point in the initial set-up of the method was the dependence of the signals on the concentration of bacteria in the sample. For the same concentrations of bacteria, the fluorescence signal was significantly more intense in comparison to the absorbance signal (which has some important benefits for testing NPs as it will be demonstrated later). Consequently, it provided a higher sensitivity in the detection of low concentrations of bacteria. On the other hand, there was a need for applying absorbance detection for higher concentrations of bacteria. Therefore, to obtain complete information of the growth process, the monitoring of the absorption and fluorescence signals was compulsory. At lower concentrations of bacteria, the fluorescence signal was lower and therefore detectable. Its comparison to the absorbance signals obtained for the same low concentrations of bacteria showed an excellent level of correlation for all three types of investigated bacteria. However, we also observed that for higher bacterial concentrations the fluorescence signal was very high and undetectable. Instead of introducing a dilution step, information on these bacteria levels was obtained from the absorption signal, which provided reliable data about bacterial growth. Therefore, to skip any additional steps while following the kinetic process (i.e., intermediate sampling, dilutions, and measurements), we performed complementary, two-signal measurements that provided a high-resolution measurement of the total 12-h kinetic process for the entire concentration range of tested bacteria.

Measurements simultaneously performed for an investigated series of eight different bacterial concentrations were used to provide a correlation between absorbance and fluorescence signals in terms of the concentration of the bacteria (Fig. 1b). As indicated in Fig. 1b, with increasing bacteria concentration, the changes in both signals have the same trend. This was confirmed for all three types of tested bacteria. Consequently, it is possible to use both detection methods, based on absorption or fluorescence (or their combination), to follow the growth kinetics of bacteria. Minimal and maximal detectable bacteria concentrations for each bacterial strain tested could be identified from Fig. 1b. Graphs are showing correlations between OD(600 nm) or fluorescence (560/590 nm) as a function of bacterial cell number (cfu/ml). The horizontal line indicates baseline corresponding to the OD/fluorescence of Presto Blue in the growth medium. Presto Blue was able to detect ≥ 10 cfu/ml of *E. coli*, > 25 cfu/ml of *P. aeruginosa* and ≥ 268 cfu/ml of *S. aureus*. Besides, direct comparison of the growth curves obtained for fluorescence and absorbance detection reveals that fluorescence saturation always appears at the same OD for the same bacterial strain. However, it is very interesting to observe that these ODs are different for different strains. For *E. coli* last detectable fluorescence signal appears at OD = 1.02 (corresponding to 3.04 × 10^8^ cfu/ml), in *P. aeruginosa* the signal appears for OD = 1.25 (corresponding to 2.54 × 10^9^ cfu/ml) and in *S. aureus* it is at OD = 1.4 (corresponding to 1 × 10^12^ cfu/ml). For higher ODs, fluorescence gets saturated and could not be detected anymore. Therefore these values of OD (and corresponding cfu/ml) are maximal concentrations of each strain of bacteria detectable using Presto Blue fluorescence detection.

**Fig. 2:**
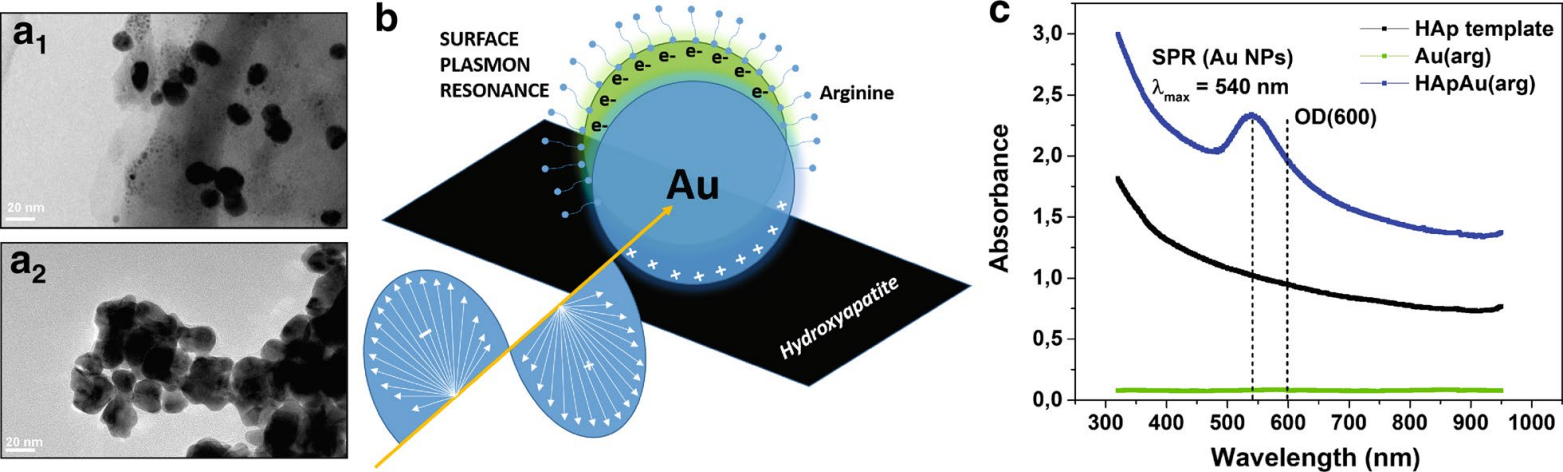
Morphological characteristics of apatite—template (**a**_**1**_) and non-templated (**a**_**2**_) Au/arginine NPs; illustration of the surface plasmon resonance (SPR) effect in the templated material with AuNPs upon illumination with the light (**b**); UV/vis absorption spectra of apatite template without metallic content, templated and non-templated Au/arginine as well as comparison of their light absorption with the wavelength frequently used for spectrophotometric monitoring of bacterial growth (OD_600_) (**c**)

In the next step, the optimized protocol was used to test bacterial growth kinetics in the presence of NPs with specific optical properties. In one particular case, we validated our method for Au NPs functionalized with amino acids on apatite template (HAp/Au/arginine) which are cationic NPs characterized by contact-based antimicrobial activity [29, 34]. Their antimicrobial activity is strongly dependant on the interactions with the bacterial cell wall and significantly depends on direct contact between NPs and bacterial surface. HAp are plated with mean values of width and length 500 nm and 100 nm, respectively and BET surface area of 24 m^2^/g [35, 36]. Within HAp/Au/arginine composites, AuNPs are 5–15 nm in size (Fig. 2a_1_) deposited at apatite plates and their BET specific surface area is 86 m^2^/g. Non-templated Au/arginine NPs are with BET specific area of 27 m^2^/g, and they aggregated structures containing 20–30-nm sized NPs (Fig. 2a_2_) within large agglomerated spheres with 500 nm in diameter [29]. When they are non-agglomerated and stably attached to the surface of the apatite template, HAp/Au/arginine NPs are characterized by the surface resonance of conduction electrons (plasmons) generated by incident light (as illustrated in Fig. 2b). Consequently, they have strong absorption at 540 nm corresponding to the surface plasmon resonance (SPR) maximum. Both components of the composite possess specific optical characteristics. Apatite is characterized by the absorption of light over the entire UV/vis region (Fig. 2c) whereas absorption by Au/arginine NPs depends on their stability (Fig. 2c). The SPR maximum of stabilized Au/arginine NPs is very close to the signal at 600 nm which is the wavelength commonly used for measuring the optical density (OD_600_, Fig. 2c) for tracking the kinetics of bacterial growth. Therefore, due to the inherent characteristics, the material has the potential to interfere with turbidity-based spectroscopic methods [37] most frequently applied for monitoring the influence of NPs on the kinetics of bacterial growth.

**Fig. 3:**
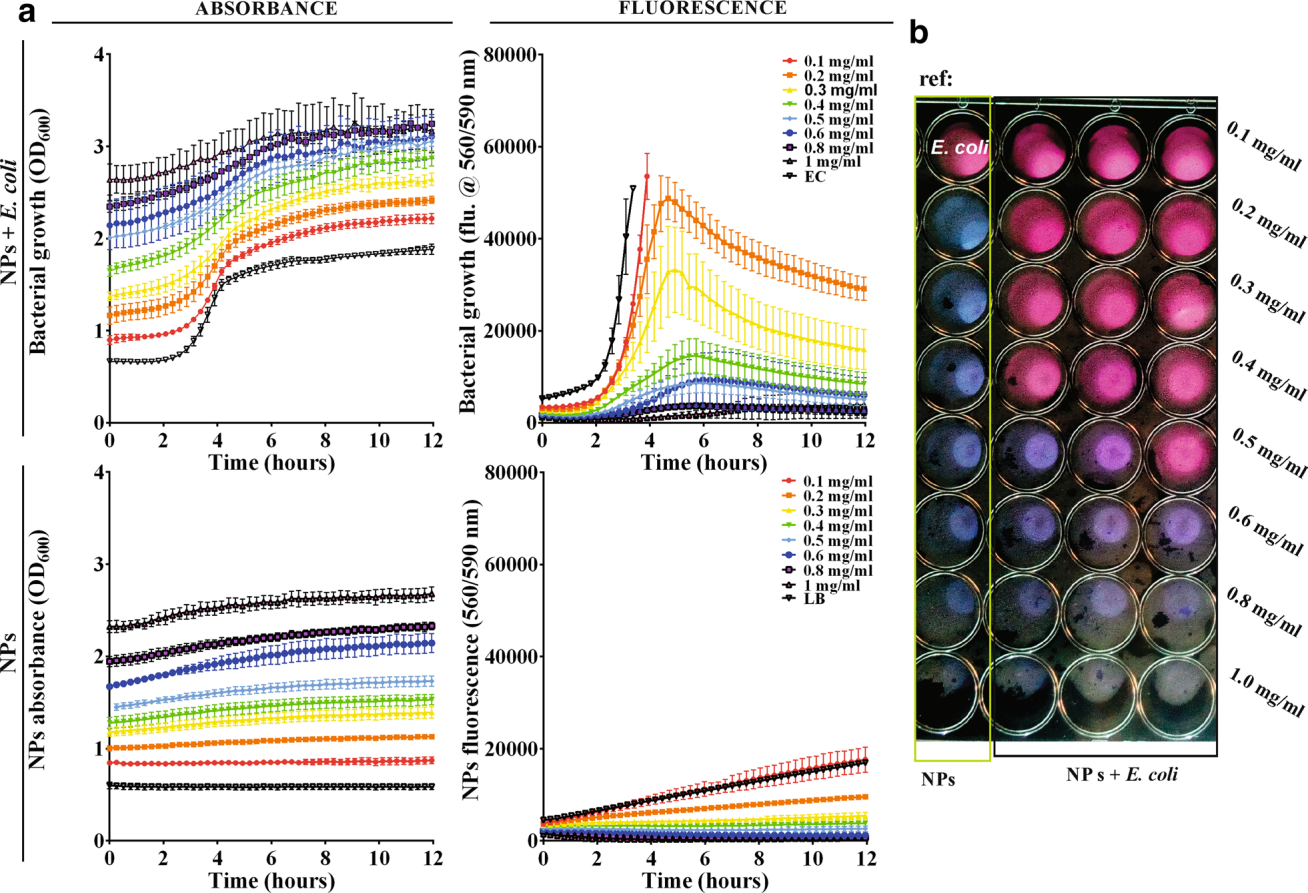
Presto Blue monitoring of the influence of HAp/Au/arginine NPs to the growth of *E. coli*: absorbance (at 600 nm) and fluorescence (at 560/590 nm) signal for the cases with and without bacteria

The capacity of this material to interfere with the turbidity-based spectroscopic detection of bacterial growth was initially confirmed by following the absorption signal at 600 nm. Gradually increasing concentrations of NPs in a LB medium (ranging from 0.1 to 1 mg/ml as indicated in the legend of Fig. 3a) without any added bacteria provided a significant signal at 600 nm (Fig. 3a). Consequently, we detected a very intense background that was even higher than the absorption signal obtained for the reference bacteria in the stationary growth phase. Due to their capacity for significantly increasing the background, NPs provided a substantial shift in the growth kinetics curves towards higher absorbance values during antimicrobial testing of the activity of the material. Moreover, all of the curves corresponding to NPs testing had the sigma-shape typical of the kinetic curves of bacterial growth. Therefore, when following the absorption signal at 600 nm only, one would conclude that NPs cannot inhibit *E. coli* growth. However, endpoints obtained after 12-h testing (Fig. 3b) clearly confirmed antimicrobial activity. Presto Blue is a blue, non-fluorescent resazurin dye (in non-metabolically active cells) which is reduced to fluorescently pink resorufin in the presence of viable bacteria [22]. During testing, similar to the pink-stained reference samples corresponding to viable bacteria, Presto Blue detected metabolic activity and turned pink at lower concentrations of NPs (up to 0.5 mg/ml). For higher concentrations (0.6–1.0 mg/ml), Presto Blue remained blue, with a color identical to that of the pure NPs without bacteria (Fig. 3b). Consequently, the endpoint reading clearly showed the absence of metabolic activity after the addition of 0.6 mg/ml as the minimal concentration of tested NPs that provided complete inhibition of bacterial growth in *E. coli* (this value corresponds to the MIC). Moreover, considering the unchanged blue color of the Presto Blue in NPs without bacteria in the reference samples, the results excluded non-specific interactions between tested NPs and Presto Blue dye. Although Au(arg) NPs have the potential to catalyze the reduction of resazurin to resorufin upon exposure to light in the presence of amines, [38] this activity was not observed during the testing. If degradation of the dye takes place, resazurin (blue, non-fluorescent) will turn into resorufin (pink, fluorescent) and if the process continues, resorufin will turn into dyhydroresorufin (colorless, non-fluorescent) [22]. Accordingly, dye degradation could be followed either by following blue-to-pink or bleaching- pink change of the color or by following appearance/quenching of the fluorescence. In general, during testing NPs, two possible sources could decompose Presto Blue dye—the light as well as a combination of NPs with the light (photocatalytic degradation). For that reason, during our study, we applied a control system containing Presto Blue with NPs in growth medium (without bacteria) at different concentrations of NPs (corresponding to those used for antimicrobial testing). The control was exposed to the light every 15 min during 12 h along with the samples used for testing antimicrobial activity (in the same 96-well plate). After the 12-h exposure, the color of the Presto Blue remained blue for the whole range of NPs concentrations (Fig. 3b, Additional file 1: Figures S1b, S2b) indicating that the resazurin that was initially added did not change. At the same time, both absorbance and fluorescence signals were low with the values similar to the control containing Presto Blue dye in growth medium (without NPs) (Fig. 3a, Additional file 1: Figures S1a, S2a). Based on these results, we concluded that Presto Blue is stable under our experimental conditions and the absence of non-specific interactions between the dye and the tested NPs were optimal for monitoring bacterial growth accurately and for testing antimicrobial activity.

In contrast to the growth curves obtained from absorption signals, the curves obtained from the fluorescence signal (excitation/emission at 560/590 nm) were much more accurate (Fig. 3a). Due to the absence of a self-fluorescent nature and the inability of NPs to interact or interfere with the dye, the fluorescence signal obtained from NPs in LB for the entire concentration range from 0.1 to 1.0 mg/ml was very low (Fig. 3a). Consequently, due to the minimal background and very good signal-to-noise ratio, the monitoring of bacterial growth kinetics through the detection of the fluorescence signal in the presence of tested NPs provided a much more realistic picture. The obtained curves clearly show a decrease in bacterial growth with increasing concentrations of NPs. For lower concentrations (0.2–0.5 mg/ml), a fraction of bacteria remained viable as detected in the pink-colored endpoints (see Fig. 3b). For higher NP concentrations (0.6–1.0 mg/ml), there is a bacteria inhibition growth (blue color). As with the endpoint viability, growth curves obtained from the fluorescence signal confirmed the value of the MIC of the investigated NPs in *E. coli* as 0.6 mg/ml. Due to a very high intensity of the fluorescence signal that reached the detectability limit, data for the growth kinetics of the reference bacteria and for the bacteria with a low concentration of NPs (0.1 mg/ml) were missing. These data were compensated by using the information obtained from the absorption signal. Growth curves obtained from the fluorescence signal for higher concentrations of NPs and growth curves obtained for lower concentrations of NPs and the reference bacterial growth provided the complete picture of the kinetics of the antimicrobial characteristics of HAp/Au/arginine NPs. Beyond the observed details regarding tracking the kinetics of bacterial growth, the accurate detection of the bacterial viability and the MIC values were also confirmed in two additional bacterial strains, *P. aeruginosa* (Additional file 1: Figure S1) and *S. aureus* (Additional file 1: Figure S2). The Presto Blue method applied to tracking the growth kinetics provided a determination of their MIC values which were between 0.4 and 0.5 mg/ml for *P. aeruginosa* and exceeded 1 mg/ml for *S. aureus*.

**Fig. 4:**
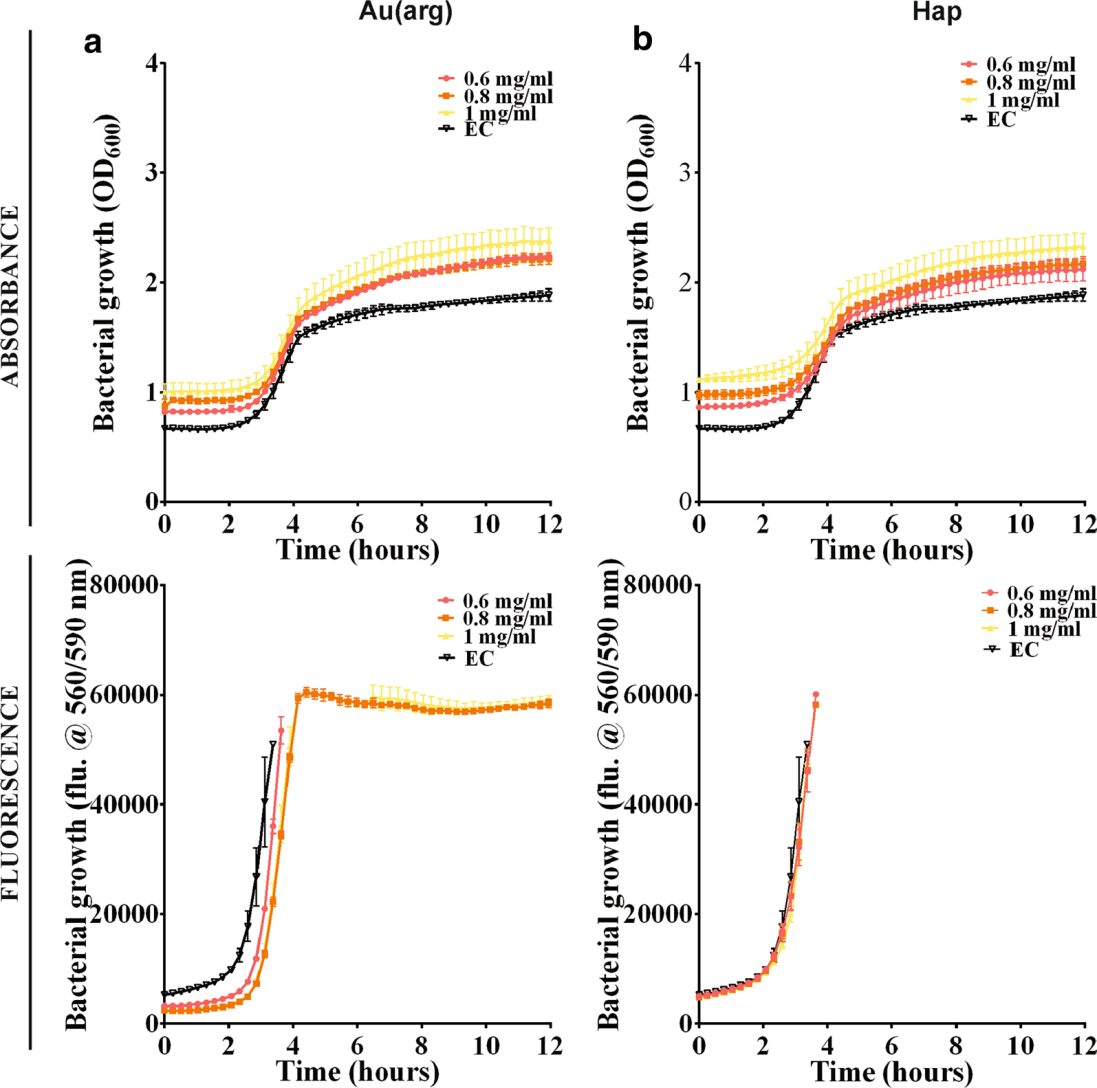
Comparison of the influence of Au(arg) (**a**) and HAp (**b**) NPs to the growth kinetics of *E. coli*: absorbance (at 600 nm) and fluorescence (at 560/590 nm) signal, respectively

The antimicrobial activity of the HAp/Au/arginine NPs was further investigated by comparing the activity of the entire structure with the activity of separated components [non-templated functionalized AuNPs (Au/arginine) and pure apatite template (Hap)] for the concentration range indicated in the legend of Fig. 4. As previously observed (Fig. 2c), the separated components and HAp/Au/arginine have different optical properties. Compared to the substantial increase of the background and the shifting of the growth kinetics to higher values of absorbance at 600 nm observed in HAp/Au/arginine, this effect was significantly lower in non-templated arginine-functionalized AuNPs (Au/arginine) and in a pure apatite template (Hap). Because they are intensely agglomerated due to the absence of stabilization provided by the apatite template (Fig. 2c) Au/arginine NPs are not characterized by an intensive SPR maximum. The maximum is also missing in the apatite absorption spectrum. Therefore, according to the absorbance signal (Fig. 2c), their contribution to the change in the bacterial growth curve is reduced. The growth curves of the *E. coli* reference and the bacteria grown with 0.6–1.0 mg/ml of Au/arginine or apatite are very similar. Based on this information, one concludes that Au/arginine NPs and Hap do not have any influence on the growth of *E. coli* as separated components. The same observations were additionally confirmed for non-templated Au/arginine and separated template tested in *P. aeruginosa* (Additional file 1: Figure S3) and *S. aureus* (Additional file 1: Figure S4) bacterial strains. Compared to the absorbance, the fluorescence signal provided higher sensitivity and confirmed that Au/arginine causes a limited decrease in the bacterial growth rate which was most pronounced for *S. aureus* (Additional file 1: Figure S4).

Presently, a range of different NPs and nanostructures are optimized to design the next generation of antimicrobials. Even more nanofeatures are involved as building components of different emerging platforms designed for innovative drugs, medical devices, and healthcare procedures of the future. Most of these structures are characterized by particular optical properties and tend to interfere with different detection and biological validation procedures. Along with the advancement of medication, there is a need for the development of medical and biological validation methods because old approaches may no longer possess the required level of accuracy. From that standpoint, the optimized Presto Blue method is a very good tool for tracking the kinetics of bacterial growth as well as the accurate detection of antimicrobial activity in NPs. This significant advancement of microbiological analytical techniques applied to the characterization of NPs is essential. It provides the possibility for very sensitive detection of bacterial growth with a very high-time resolution and the excellent stability required for long-term examinations (including the dynamic process). In particular case of HAp/Au/arginine NPs, the method provided accurate tracking of the influence of these NPs to bacterial strains characterized with very specific membrane compositions [39–41]. Since electrostatic interactions among arginine-functionalized NPs and bacterial cell wall are critical for their antibacterial activity, it revealed an exciting, new direction for future investigations regarding the interactions existing at the interface between the bacterial wall and cationic Au/arginine NPs. In general, we believe that this method can be tailored for other detection purposes when the optical properties of NPs and nanostructures are limiting factors in resolving a number of other issues hidden within the high background caused by non-specific interactions of NPs.

## Conclusions

The resazurin-based (Presto Blue) method with the combined detection of absorbance and fluorescence signals is highly applicable for accurate tracking of bacterial growth kinetics and the sensitive detection of how bacterial viability is affected by NPs with specific optical characteristics (functionalized AuNPs). The absorbance signal provides useful data for following bacterial growth with and without very low concentrations of NPs. On the other hand, the fluorescence signal provides bacterial growth data in the presence of higher concentrations of NPs which affects the kinetics of bacterial growth. In these cases, the signal-to-noise ratio was optimal, so it could be effectively used for sensitive and high-time resolution tracking of bacterial growth and detection of bacterial viability. The optimized method is tailorable for a range of NPs with specific optical characteristics and for tracking their influence on bacterial growth, thus properly evaluating their antibacterial activities.

## Methods

### Materials

Calcium nitrate tetrahydrate (≥ 99.0%) [Ca(NO_3_)_2_·4H_2_O (Sigma-Aldrich, Germany)], ammonium dihydrogen phosphate (99.999% trace metals basis) [NH_4_H_2_PO_4_ (Sigma-Aldrich, Germany)] and urea (≥ 98%) [(NH_2_)_2_CO, (Alfa Aesar, Germany)] were used for homogeneous precipitation of apatite (Ca_5_(PO_4_)_3_OH). The Au(arg) NPs and their composites with apatite were prepared with chloroauric acid (~ 52% Au basis) [HAuCl_4_ (Sigma-Aldrich, Germany)]. The chemical reduction was performed using l-arginine amino acid (≥ 98%) (l-2-amino-5-guanidinopentanoic acid; C_16_H_14_N_4_O_2_), (Sigma-Aldrich, Germany). All chemicals and reagents were of analytical grade. All experiments were performed using lab-produced, ultra-distilled water.

### Methods for synthesis and functionalization of NPs

#### Synthesis Au/arginine NPs

The synthesis of the Au(arg) NPs was performed using chemical reduction [29, 34]. 50 ml of water was pre-treated by ultrasonication (VCX 750, Connecticut, USA) (time of sonication t = 10 min, pulsation-to-relaxation periods on:off = 02:01 s, T = 25 °C, power P = 600 W, frequency f = 20 kHz, and amplitude A = 80%). A total volume of 1 ml of isopropanol was used to intensify the sonication process. Afterwards, 50 ml of an aqueous solution of HAuCl_4_ (0.8 mg/ml) and l-arginine (0.4 mg/ml) was added and the sonication was continued for 30 min using the same parameters. The precipitate obtained was separated from the supernatant by centrifugation (15 min at 4100 rpm) and air dried.

#### Synthesis apatite

Apatite was synthetized using sonochemical homogeneous precipitation [35, 42]. 50 ml of aqueous solutions of Ca(NO_3_)_2_·5H_2_O and NH_4_H_2_PO_4_ were mixed to obtain Ca:P ratio 1:2 wt%. The mixture was pre-heated to 80 °C when 10 ml of urea (12 wt%) was added. After reaching the required temperature, sonication was started and it was performed during the next 3 h using the following parameters: pulsation-to-relaxation periods on:off = 02:01 s, power P = 600 W, frequency f = 20 kHz, and amplitude A = 80% (VCX 750, Connecticut, USA). Sonication and heating induced the decomposition of urea into ammonium products that gradually increased the pH and induced the precipitation of calcium phosphate which was transformed into apatite during the aging of the precipitate in the supernatant for the next 24 h. After aging, the precipitate was centrifuged (10 min at 4000 rpm) to remove the supernatant and washed twice with distilled water.

#### Synthesis HAp/Au/arginine

Composite was formed by combining chemical reduction with homogeneous precipitation [29, 34]. Sonochemically synthesized apatite (washed and non-dried) was re-dispersed in 50 ml of water (1.5 mg/ml) using ultrasonication (time of sonication t = 10 min, pulsation-to-relaxation periods on:off = 02:01 s, T = 25 °C, power P = 600 W, frequency f = 20 kHz, and amplitude A = 80%) (VCX 750, Connecticut, USA). A total volume of 1 ml of isopropanol was used to intensify the sonication process. After the dispersion period was finished, 50 ml of an aqueous solution of HAuCl_4_ (0.8 mg/ml) and l-arginine (0.4 mg/ml) was added and the sonication was continued for 30 min using the same parameters.

### Methods for characterization of NPs

Morphological and structural investigations of non-templated Au/arginine NPs and HAp/Au/arginine NPs stabilized with a template were performed using transmission electron microscopy (JEOL JEM-2100). The optical properties of NPs were analyzed using UV/vis spectrophotometry (UV–vis-NIR spectrophotometer Shimadzu UV-3600). Spectra were acquired between 300 and 800 nm with a resolution of 0.1 nm.

### Method for Presto Blue kinetic study

The method was initially optimized in three different bacterial strains—two Gram-negative [*E. coli* MG1655 (ATCC 47076) and *P. aeruginosa* PAO1 (ATCC 15692)] and a Gram-positive [*S. aureus* Rosenbach (ATCC 12600)]. The strains were cultured overnight at 37 °C in Luria Bertani (LB) (pH = 7.2 ± 0.2) (Scharlab, Spain) liquid medium for *E. coli* and *P. aeruginosa* and in tryptic soy broth (TSB) (pH = 7.3 ± 0.2) (Scharlab, Spain) medium for *S. aureus*. Bacterial growth in an overnight culture was quantified by measuring the optical density at 600 nm (OD_600_). The overnight cultures were diluted to an optical density (OD) of 1 which was used as a stock to obtain a series of bacterial dilutions ranging from 10^−1^ to 10^−8^. The test was performed by adding 180 μl of bacteria with a specific OD into the wells of a 96-well assay plate (tissue culture-treated polystyrene; Costar 3595, Corning Inc., Corning, NY) in four different replicates. Then, 20 μl of Presto Blue™ Cell Viability Reagent (10×) (Molecular Probes, Invitrogen, Thermo Fisher Scientific) were added and intensive mixing with a micropipette was performed. The growth medium (LB or TSA) containing 20 μl of Presto Blue was used as a reference. Microplate incubation was performed in an Infinite M200 Pro multimode microplate reader (Tecan). The bacteria were incubated at 37 °C with an orbital shaking of 4-mm amplitude for 12 h. Every 15 min the absorbance signal was read at 600 nm followed by a reading of the fluorescence signal with emission/excitation at 560/590 nm. After a 12-h incubation, the bacteria were serially diluted in PBS (Fisher BioReagents, pH = 7.4 ± 0.1; 11.9 mM phosphates, 137 mM NaCl and 2.7 mM KCl) and 50 μl of each dilution was plated on LB or TSA plates to count the colony forming units (cfu) for the correlation of the endpoint absorbance and fluorescence signals obtained during the bacterial growth kinetic study.

### Method for the Presto Blue kinetic study in NPs

The antibacterial activity of the NPs was performed for Au/arginine, apatite, and HAp/Au/arginine. All three types of NPs were ultrasonically dispersed in bacterial growth media (LB or TSB) for 30 s (A = 18%, W = 250 W, on:off = 2:1 s) to form 2 mg/ml stocks. Microtiter plate wells were inoculated with 100 μl of bacteria (OD = 0.05) followed by the addition of serial dilutions of the specific NPs (concentration range from 0.1 to 1.0 mg/ml). A 20-μl aliquot of Presto Blue was added to each well in the final step. The controls included the growth medium (LB or TSB) with Presto Blue, bacteria (OD = 0.05) in the growth medium along with Presto Blue (without NPs) and finally, NPs (for each tested concentration) in the growth medium combined with Presto Blue (without bacteria). All concentrations were tested in four replicates.

## Additional file

**Additional file 1.** **Figure S1.** Presto Blue monitoring of the influence of HApAu(arg) NPs to the growth of *P. aeruginosa*: optical density (at 600 nm) and fluorescence signal (at 560/590 nm) for the cases with and without bacteria. **Figure S2.** Presto Blue monitoring of the influence of HApAu(arg) NPs to the growth of *S. aureus*: optical density (at 600 nm) and fluorescence signal (at 560/590 nm) for the cases with and without bacteria. **Figure S3.** Comparison of the influence of Au(arg) (a) and HAp (b) NPs to the growth kinetics of *P. aeruginosa*: absorption (at 600 nm) and fluorescence (at 560/590 nm) signal, respectively. **Figure S4.** Comparison of the influence of Au(arg) (a) and HAp (b) NPs to the growth kinetics of *S. aureus*: optical density (at 600 nm) and fluorescence signal (at 560/590 nm), respectively.

## Abbreviations

NPs: nanoparticles
OD_600_: optical density at 600 nm
SPR: surface plasmon resonance
MIC: minimal inhibitory concentration
MBC: minimal biocidal concentration
LB: Luria–Bertani broth
TSB: tryptic soy broth
