# Supplementary material for: High time resolution and high signal-to-noise monitoring of the bacterial growth kinetics in the presence of plasmonic nanoparticles
Source: J Nanobiotechnology. 2019 Feb 1;17:21. doi: 10.1186/s12951-019-0459-1 (PMC6357367; doi:10.1186/s12951-019-0459-1)
Supplement: Supplementary file 1 — Additional file 1. Figure S1. Presto Blue monitoring of the influence of HApAu(arg) NPs to the growth of P. aeruginosa: optical density (at 600 nm) and fluorescence signal (at 560/590 nm) for the cases with and without bacteria. Figure S2. Presto Blue monitoring of the influence of HApAu(arg) NPs to the growth of S. aureus: optical density (at 600 nm) and fluorescence signal (at 560/590 nm) for the cases with and without bacteria. Figure S3. Comparison of the influence of Au(arg) (a) and HAp (b) NPs to the growth kinetics of P. aeruginosa: absorption (at 600 nm) and fluorescence (at 560/590 nm) signal, respectively. Figure S4. Comparison of the influence of Au(arg) (a) and HAp (b) NPs to the growth kinetics of S. aureus: optical density (at 600 nm) and fluorescence signal (at 560/590 nm), respectively. [file 12951_2019_459_MOESM1_ESM.docx]

**Additional file 1**

**High Time Resolution and High Signal-to-Noise Monitoring of the Bacterial Growth Kinetics in the Presence of Plasmonic Nanoparticles**

Marija Vukomanovic*^,1,2^ Eduard Torrents*^,1^

^1^Bacterial Infections: Antimicrobial Therapies, Institute for Bioengineering of Catalonia (IBEC), The Institute of Science and Technology, Baldiri Reixac 15-21, 08028 Barcelona, Spain

[mvukomanovic@ibecbarcelona.eu](mailto:mvukomanovic@ibecbarcelona.eu), [etorrents@ibecbarcelona.eu](mailto:etorrents@ibecbarcelona.eu)

^2^Advanced Materials Department, Institute Jozef Stefan, Jamova 39, Ljubljana, Slovenia

[marija.vukomanovic@ijs.si](mailto:marija.vukomanovic@ijs.si)

*Corresponding authors

**Figure S1.** Presto Blue monitoring of the influence of HApAu(arg) NPs to the growth of *P. aeruginosa*: optical density (at 600 nm) and fluorescence signal (at 560/590 nm) for the cases with and without bacteria.

**Figure S2.** Presto Blue monitoring of the influence of HApAu(arg) NPs to the growth of *S. aureus*: optical density (at 600 nm) and fluorescence signal (at 560/590 nm) for the cases with and without bacteria.

**Figure S3.** Comparison of the influence of Au(arg) (a) and HAp (b) NPs to the growth kinetics of *P. aeruginosa*: absorption (at 600 nm) and fluorescence (at 560/590 nm) signal, respectively.

**Figure S4.** Comparison of the influence of Au(arg) (a) and HAp (b) NPs to the growth kinetics of *S. aureus*: optical density (at 600 nm) and fluorescence signal (at 560/590 nm), respectively.
